# Oscillatory neural network learning for pattern recognition: an on-chip learning perspective and implementation

**DOI:** 10.3389/fnins.2023.1196796

**Published:** 2023-06-15

**Authors:** Madeleine Abernot, Nadine Azemard, Aida Todri-Sanial

**Affiliations:** ^1^Laboratoire d'Informatique de Robotique et de Microélectronique de Montpellier (LIRMM), Department of Microelectroncis, University of Montpellier, CNRS, Montpellier, France; ^2^Electrical Engineering Department, Eindhoven University of Technology, Eindhoven, Netherlands

**Keywords:** oscillatory neural networks, on-chip learning, unsupervised learning, pattern recognition, FPGA implementation

## Abstract

In the human brain, learning is continuous, while currently in AI, learning algorithms are pre-trained, making the model non-evolutive and predetermined. However, even in AI models, environment and input data change over time. Thus, there is a need to study continual learning algorithms. In particular, there is a need to investigate how to implement such continual learning algorithms on-chip. In this work, we focus on Oscillatory Neural Networks (ONNs), a neuromorphic computing paradigm performing auto-associative memory tasks, like Hopfield Neural Networks (HNNs). We study the adaptability of the HNN unsupervised learning rules to on-chip learning with ONN. In addition, we propose a first solution to implement unsupervised on-chip learning using a digital ONN design. We show that the architecture enables efficient ONN on-chip learning with Hebbian and Storkey learning rules in hundreds of microseconds for networks with up to 35 fully-connected digital oscillators.

## 1. Introduction

Current Artificial Intelligence (AI) models are mainly used for two functions, overcoming the human brain to solve a specific task, or replacing the human brain on more general purpose tasks (Pehlevan and Chklovskii, 2019). In both cases, AI models need to learn how to correctly solve a given task. However, while humans are capable of learning continuously through life to adapt to the changing environment and learn new tasks, current AI models are trained in advance for inference, making it impossible to learn from evolving environments and input data (Thrun and Mitchell, 1995; Ring, 1997). To adapt AI models to evolving environments and input data, continual learning is necessary, so there are ongoing efforts to develop continual learning algorithms for AI models (Thangarasa et al., 2020). In particular, efforts are concentrated first on supervised continual learning (De Lange et al., 2022; Mai et al., 2022) to improve the performance of classification models over time, and then on continual reinforcement learning to learn from the environment, for example in robotics (Lesort et al., 2020; Khetarpal et al., 2022).

Continual learning algorithms expect to learn novel data while avoiding catastrophic forgetting (McCloskey and Cohen, 1989; French, 1999) of previously learned data, for example, considering bio-inspired synaptic plasticity, or reminding solutions (Hayes et al., 2020; De Lange et al., 2022; Jedlicka et al., 2022). Additionally, continual learning demands to be implemented on-chip for fast and efficient performances. However, to allow continual on-chip learning, each synapse needs to be re-programmable in a real-time latency requiring additional space, and resources, and consuming more energy consumption than systems without on-chip learning.

Moreover, there are several ongoing works to propose hardware implementations of fast, low-resource, and power-efficient AI computing paradigms. In particular, neuromorphic computing (Christensen et al., 2022) takes inspiration from the human brain neural network for the AI models architectures, and for the data representation. The most widely used neuromorphic computing paradigm is called Spiking Neural Network (SNN; Maass, 1997) which takes inspiration from spikes transmitted among neurons through the brain synapses by encoding information in the latency between two spike signals. SNN has been widely explored in the last decades both in terms of network implementation, with the development of different SNN-based chips for edge AI computing (Davies et al., 2018; Pehle et al., 2022), and in terms of learning, in particular for continual learning (Wang et al., 2014; Lobo et al., 2019). In this paper, we focus on another neuromorphic paradigm, called the Oscillatory Neural Network (ONN), which is drawing attention as an alternative neuromorphic solution for edge AI computing.

ONN takes inspiration from the collective synchronization of human brain neurons through oscillations (Tognoli and Kelso, 2009). ONN is an analog-based computing paradigm built as a network of coupled oscillators (Izhikevich and Kuramoto, 2006; Schwemmer and Lewis, 2012; Raychowdhury et al., 2019; Csaba and Porod, 2020; Todri-Sanial et al., 2022) computing with the parallel phase synchronization of coupled oscillators, called phase computing. In phase computing, information is encoded in the phase relationship between oscillators which can potentially limit voltage amplitude and, therefore, reduce the energy consumption (Delacour et al., 2023a), making it attractive for edge computing. Currently, efforts are given on ONN implementation, from materials to devices, on ONN circuit architecture (Abernot et al., 2021; Delacour et al., 2023b), and on ONN applications with demonstrators of ONNs for image processing (Fernandes et al., 2004; Abernot and Todri-Sanial, 2023), robotic navigation (Abernot et al., 2022a), or optimization problems (Wang and Roychowdhury, 2019; Delacour et al., 2022). Yet, learning and continual learning algorithms for ONN are still to be investigated. Thus, this work focuses on ONN on-chip learning for pattern recognition.

**Figure 1:**
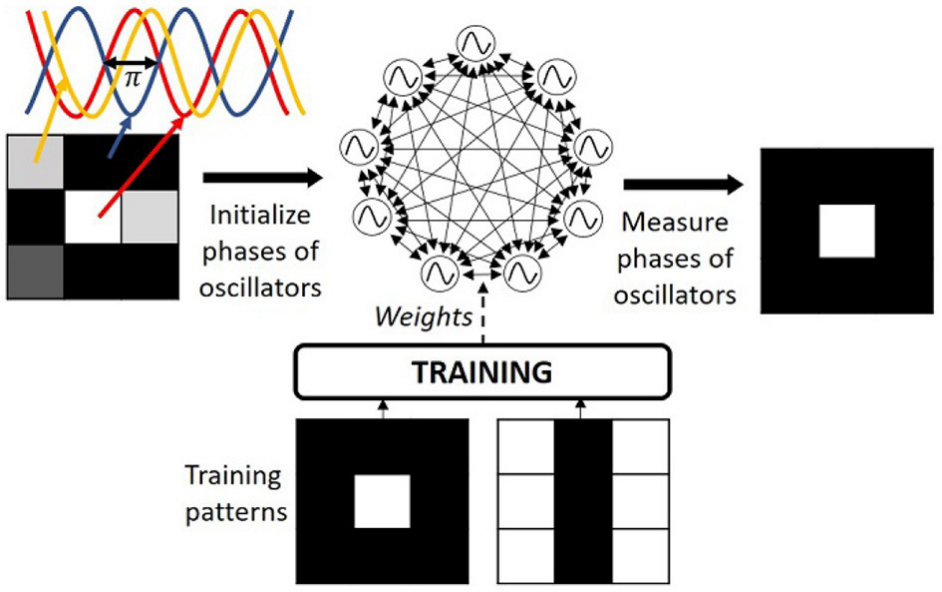
ONN computing paradigm configured for pattern recognition.

In state-of-the-art, ONNs are often studied as a fully-connected recurrent architecture to perform pattern recognition similar to Hopfield Neural Networks (HNNs) (Hoppensteadt and Izhikevich, 1997; Nikonov et al., 2015; see Figure 1). While in the literature ONNs are typically trained with unsupervised learning rules that were first introduced for HNNs. To the best of our knowledge, learning rules specific to ONNs are yet to be developed. In this work, we present an adaptation of HNN unsupervised learning rules for ONNs while analyzing the different learning rules for continual on-chip learning. Recently, we introduced an on-chip learning architecture for a digital ONN implementation (Abernot et al., 2022b) with the Hebbian learning rule applied to a small 15-neuron ONN for a three-digit pattern recognition application. In this work, we go beyond by demonstrating that the ONN architecture is compatible with other learning rules than Hebbian by implementing the Storkey learning rule. Next, we analyze the scalability of the ONN architecture to provide a more complete evaluation of the system.

The main contributions of the paper are summarized as (i) adaptation of existing HNN unsupervised learning rules to ONNs, (ii) development of a continual on-chip learning algorithm on ONN with unsupervised learning rules, (iii) an implementation approach for on-chip learning on digital ONN for auto-associative memory tasks, and (iv) present a scalability analysis of our approach in terms of latency, precision and resource utilization.

First, Section 2.1 presents the ONN paradigm and its auto-associative memory capabilities. Then, Section 2.2 gives details on the various learning rules introduced for HNN and their compatibility with ONN for on-chip learning. After, Section 2.3 defines the proposed hardware implementation to perform on-chip learning with a digital ONN design. Section 3 shows results obtained with our on-chip learning solution for various ONN sizes, learning algorithms, and weight precision. Finally, Section 4 discusses the results compared to state-of-the-art and the advantages and limitations of our on-chip learning implementation.

## 2. Materials and methods

### 2.1. Oscillatory neural networks

In ONNs, each neuron is an oscillator coupled with synaptic elements representing weights between neurons (Delacour and Todri-Sanial, 2021), and information is represented in the phase relationship between oscillators such that ONN computes in phase using the weakly coupled oscillator dynamics (Schwemmer and Lewis, 2012). For example, for binary information, if an oscillator oscillates with a 0° phase difference from a reference oscillator, it will represent a binary “0” value, while if it oscillates with a 180° phase difference from a reference oscillator, it will represent a binary “1” value. Typically, one oscillator from the network is used as the reference oscillator. The inference process starts with the initialization of each neuron phase as the input information, then, oscillators' phases evolve in parallel thanks to the dynamics of coupled oscillators (Schwemmer and Lewis, 2012) until stabilization to a final phase state, which represents the ONN inference output (see Figure 1). Phase computation can potentially reduce the voltage amplitude meanwhile it enables parallel computation, providing an attractive low-power edge computing paradigm (Delacour et al., 2023a).

**Figure 2:**
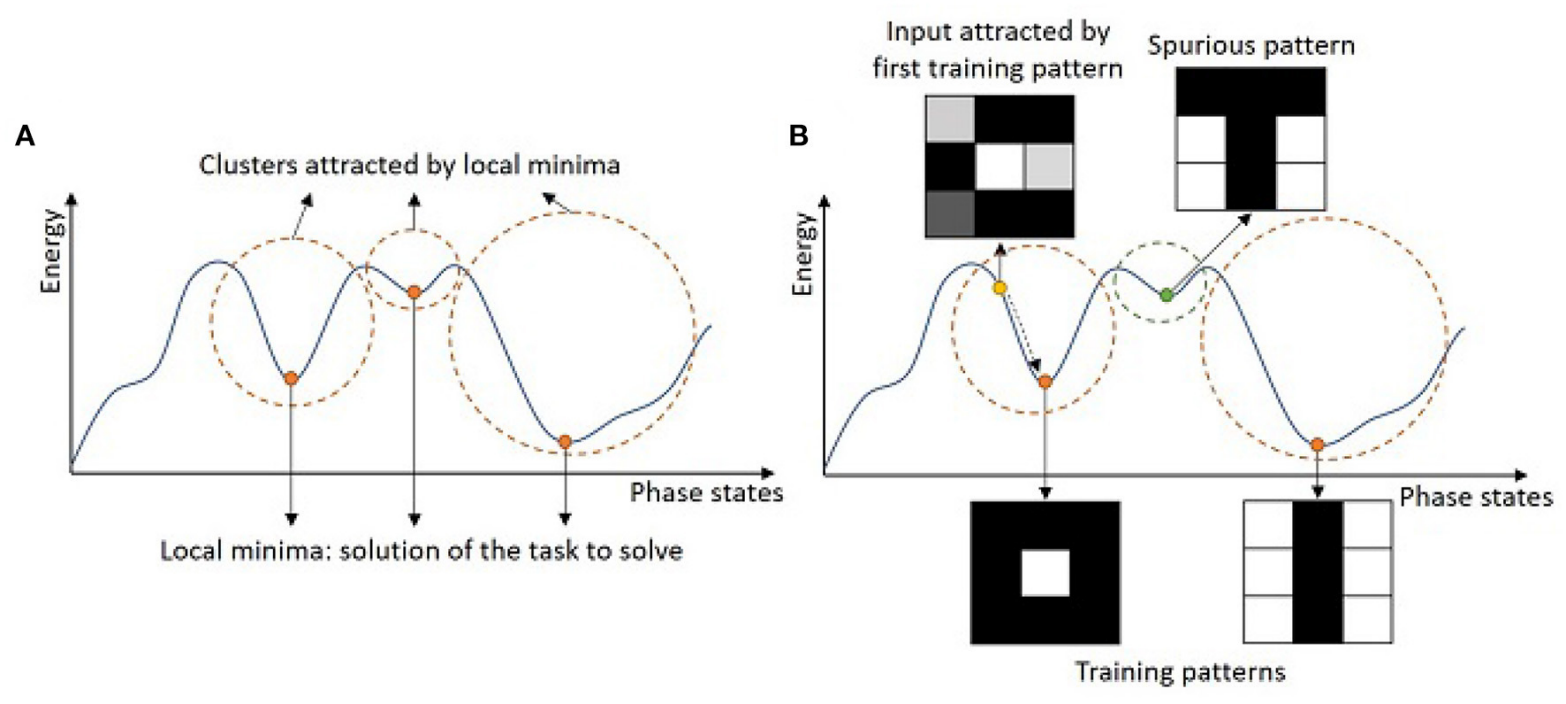
Simplified representation of an energy landscape for **(A)** a global interpretation, and **(B)** an interpretation in the case of pattern recognition.

The evolution of the phases during inference is associated with the minimization of an intrinsic parameter called the energy of the network. Note, it does not have any relationship with the power consumption of the system. The energy of the network is defined as follows:


(1) $$\begin{array}{l} E=\underset{i}{\sum }\underset{j}{\sum }W_{ij}\phi_{i}\phi_{j} \end{array}$$

with *ϕ*_*i*_ the phase state of neuron *i*, *ϕ*_*j*_ the phase state of neuron *j*, and *W*_*ij*_ the coupling weight between neuron *i* and neuron *j*. Considering this intrinsic energy parameter, ONN learning consists of shaping the energy function, and more importantly, defining the minima of this energy function given a specific task (see Figure 2A). For example, ONN can solve graph optimization problems, like max-cut (Bashar et al., 2020; Delacour et al., 2022, 2023b; Vaidya et al., 2022), graph coloring (Wang and Roychowdhury, 2019), or traveling salesman problem (Landge et al., 2020), by mapping a graph to an ONN such that if you start the ONN with random phases, it will evolve to the optimal solutions represented by the minima of the energy function. More commonly, ONN is used to solve auto-associative memory, or pattern recognition tasks (Hoppensteadt and Izhikevich, 1997; Nikonov et al., 2015) using a fully-connected architecture as in HNNs (Hopfield, 1982; see Figure 1). Interestingly, the energy function is shaped such that training patterns are minima of the energy landscape (see Figure 2B), and when the network starts on corrupted information, it will evolve and stabilize to one of the training patterns. Note, for simplicity, we represent the energy function as a two-dimensional function, however, it is N-dimensional depending on the states of the *N* neurons.

### 2.2. ONN on-chip learning for pattern recognition

In this paper, we focus on auto-associative memory tasks or pattern recognition. The pattern recognition task is first defined, then the ONN learning is presented. Finally, we explain constraints, adaptation, and compatibility of unsupervised learning rules for use as on-chip learning on ONN.

#### 2.2.1. Pattern recognition

In this work, we define the pattern recognition task, also called the auto-associative memory task, as the ability to learn patterns and retrieve them from corrupted input information. For example, considering images as patterns, a system configured for pattern recognition can memorize images and retrieve them from corrupted input with noisy or missing pixels. Classical HNNs are fully connected recurrent networks, also characterized by an energy function, which are state-of-the-art neural networks for solving pattern recognition (Hopfield, 1982). In classical HNN, each neuron follows a *sign* activation function, allowing two bipolar activation values {−1;1}, where in the case of images, each neuron represents a pixel, and the neuron activation value {−1} or {1} represents the pixel color. Thus, classical HNN can treat and learn binary patterns, like images with black and white pixels. Recently, alternative HNNs are proposed to treat and learn multi-state or continuous patterns, such as the complex HNN using complex activation functions and complex weights (Muezzinoglu et al., 2003; Tanaka and Aihara, 2009), or the modern HNN considering a *softmax* activation function (Ramsauer et al., 2021). For ONNs, each neuron activation can take various phase values depending on the ONN design such as for the treatment of multi-state or continuous information, like gray-scale images.

For pattern recognition, the couplings among neurons represent the memory of the network. During the learning process, the training algorithm defines the coupling weight values such that learning patterns become the minima on the energy landscape. Learning does not ensure that all local minima are training patterns, and in some cases, local minima can become stable phase states while it does not correspond to any learning pattern, which is also labeled as a spurious pattern (see Figure 2B). During the inference process, one input pattern is applied to the network by initializing the oscillators' phases with the corresponding input information. Then, phases evolve thanks to the inherent phase interaction between coupled oscillators until they stabilize and the final phase state represents the ONN output pattern (see Figure 1).

#### 2.2.2. ONN learning for pattern recognition

Existing learning algorithms to train an ONN for pattern recognition are mainly unsupervised learning rules, which were first introduced for HNNs. Unsupervised learning algorithms only use learning patterns to compute coupling weights, without additional feedback, unlike supervised learning algorithms, and are mainly used to solve clustering problems. In pattern recognition, each pattern becomes the point of attraction of various clusters created from the energy landscape (see Figure 2A). In this section, we discuss how to adapt HNN-based unsupervised learning algorithms for ONN.

Adapting HNN unsupervised learning rules to ONN requires weight matrix symmetry and zero diagonal values to avoid self-coupling. Originally, in HNN, the weight matrix is symmetric, meaning weights between two neurons in both directions have the same values, and the weight matrix diagonal has zero values to avoid self-coupling. Later, to improve precision and capacity, novel unsupervised learning algorithms were introduced allowing asymmetric weight matrix (Diederich and Opper, 1987; Krauth and Mezard, 1987; Gardner, 1988) and self-coupling (Gosti et al., 2019). However, most ONN implementations, in particular analog ONN implementations, do not support self-coupling and non-symmetric weights as the coupling is often implemented with discrete analog components like resistors or capacitors (Delacour and Todri-Sanial, 2021). Consequently, even if the digital ONN supports non-symmetric weights and self-coupling, there are ongoing efforts to develop alternative analog ONN designs to allow self-coupling and non-symmetric weights (Delacour et al., 2023b). Most unsupervised learning algorithms introduced for HNN can be modified to be used with ONNs by adding constraints on the weight matrix. However, it was shown to impact negatively the HNN precision and memory capacity (Tolmachev and Manton, 2020). We provide a classification of the unsupervised learning rules respecting weights symmetry and 0-diagonal in Section 3. Moreover, using unsupervised learning algorithms introduced for classical HNN limits patterns to binary information while ONN with its continuous phase values could, in principle, stabilize to non-binary patterns e.g., any phase between 0° and 360°. However, to the best of our knowledge, there exist no unsupervised learning rules for pattern recognition adapted to ONN capable of learning non-binary patterns.

#### 2.2.3. ONN on-chip learning adaptation

In this work, we define ONN on-chip learning for pattern recognition as the ability of an ONN-computing system to learn new patterns by updating ONN coupling weights meanwhile avoiding catastrophic forgetting of previously memorized patterns.

There exist mainly two features to categorize unsupervised learning rules for pattern recognition: locality which means that the update of the coupling weight between neuron *i* and neuron *j* only depends on activation values of neurons *i* and *j* on both sides of the synapse, and incrementality, which means that the update of the weights can be done pattern by pattern without forgetting previously learned patterns. The locality feature is important for on-chip learning because the update of the weights can be implemented by using limited additional resources in each synapse. Though locality is not mandatory as the update of the weights is not always integrated and implemented at the synapse level. The incrementality feature is also important to be able to learn patterns one at a time. For efficient incremental learning, previously learned patterns are memorized in the weight matrix of the network to avoid learning them again. To avoid catastrophic forgetting, some algorithms require repetitive learning of previous and novel patterns but it is not optimal for on-chip learning as it requires additional computing, and memory (Personnaz et al., 1986; Diederich and Opper, 1987; Krauth and Mezard, 1987; Gardner, 1988). Adding learning capacity to every synapse can be costly in terms of resources, so it is important to also consider sparsity and weight precision in the weight matrix. In this work, we study the impact of weight precision on HNN and ONN performances.

### 2.3. On-chip learning architecture

Here, we propose an architecture to perform ONN on-chip learning for pattern recognition. In particular, we consider a digital ONN implementation on FPGA, introduced in Abernot et al. (2021) and we explore its capability for on-chip learning. The on-chip learning architecture was first introduced in Abernot et al. (2022b) for a small-size ONN with 15 neurons, however, in this work, we study architecture scalability for different ONN sizes, learning rules, and weight precision. Here, we present the digital ONN design implementation for pattern recognition, its adaptation to on-chip learning, and our evaluation methods.

#### 2.3.1. Digital ONN design

ONNs with their phase dynamics are intrinsically analog in nature and implemented with analog computing for low-power implementations (Delacour et al., 2023a). However, digital ONNs are attractive implementations for studying various applications, fast demonstration, and investigating scalability (Moy et al., 2022; Lo et al., 2023). In particular, a digital ONN implementation on FPGA was introduced in Abernot et al. (2021) to explore novel ONN architectures, learning algorithms, and applications. The digital ONN on FPGA showcased fast and efficient computation for edge applications, for example performing obstacle avoidance on mobile robots by reading proximity sensor information (Abernot et al., 2022a), replacing convolution filters for image edge detection (Abernot and Todri-Sanial, 2023), or even accelerating the SIFT feature detection algorithm (Abernot et al., 2023a). We believe ONN implementation on FPGA is attractive for real-time applications for which providing on-chip learning is important.

**Figure 3:**
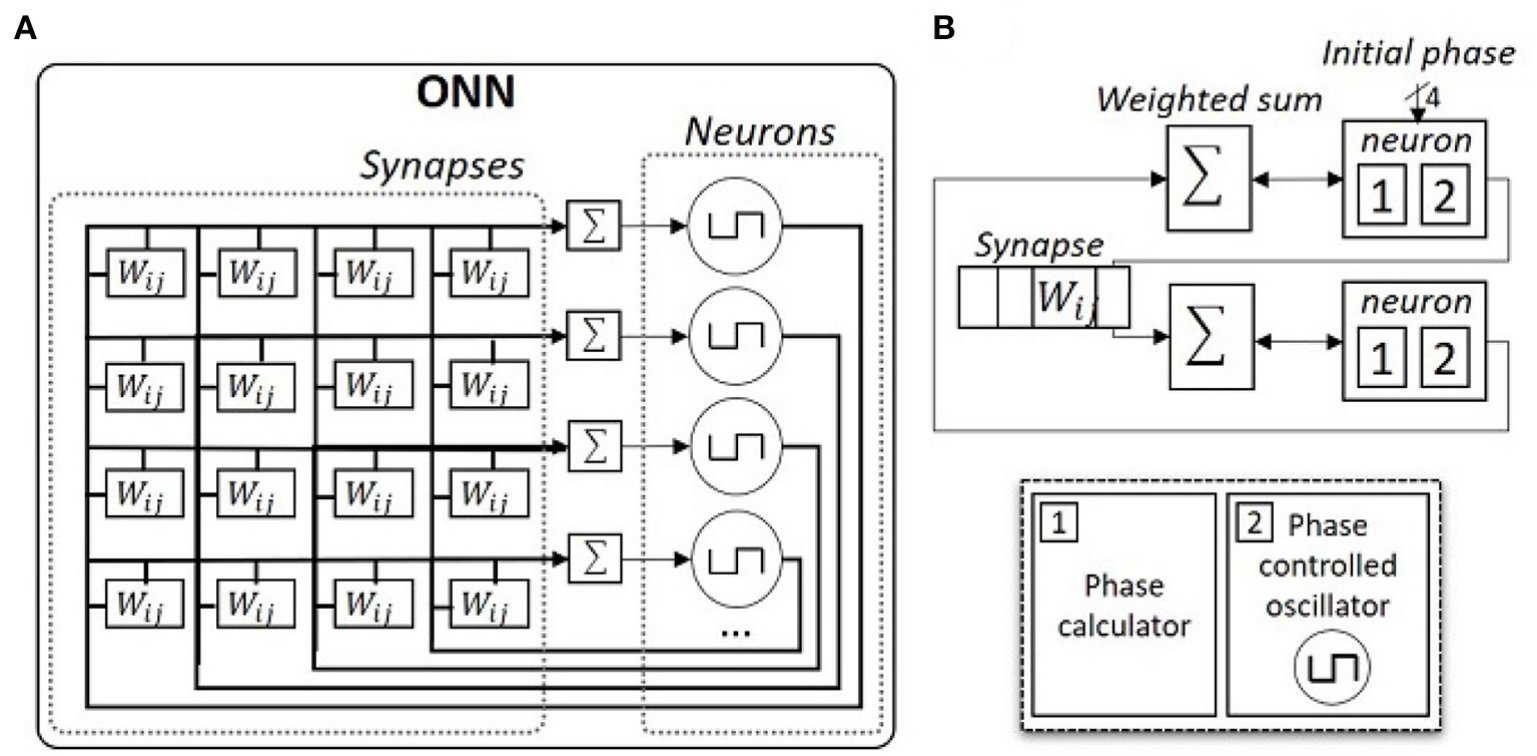
Schematics of ONN digital design. **(A)** Schematic of a fully-connected digital ONN. **(B)** Detailed schematic of a two-neuron digital ONN.

Hence, we focus on the digital ONN implementation on FPGA as introduced in Abernot et al. (2021). In the digital design, each neuron is a 16-stage phase-controlled digital oscillator that can represent phases between 0 and 180° with a precision of 22.5° and each synapse is implemented using signed registers (see Figure 3). Originally, synapses are fixed to 5-bit signed registers, but in this work, we study the impact of weight precision on resource utilization, precision, and latency of the ONN on-chip learning architecture. We especially test three weight precision, with 3-, 4-, and 5-bit signed register implementations. Note, the digital design allows the implementation of non-symmetric weights with self-coupling (non-zero diagonal). However, in this work, we only consider symmetric weights without self-coupling to be coherent and compatible with other ONN implementations, for example, analog ONN designs (Jackson et al., 2018; Moy et al., 2022).

#### 2.3.2. Architecture for on-chip learning

**Figure 4:**
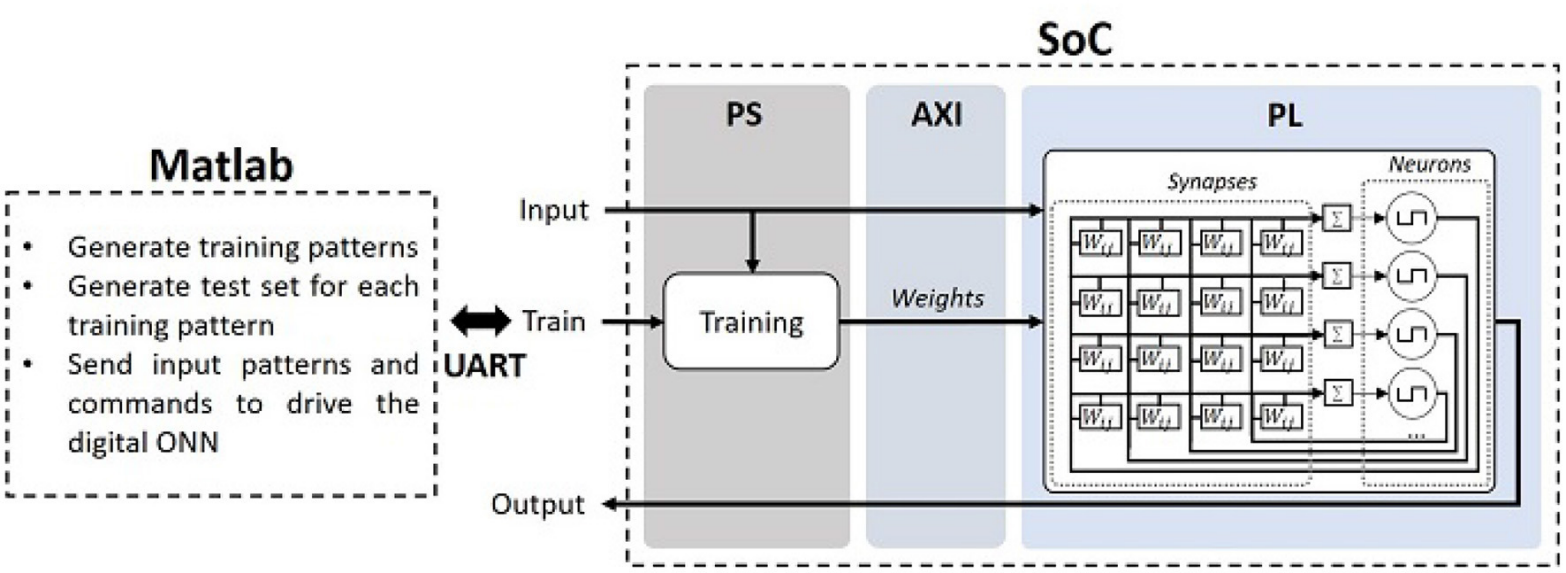
Architecture for ONN on-chip learning.

In this work, we perform ONN on-chip-learning using the digital ONN design in an architecture implemented on the Zybo-Z7 development board (Digilent, 2018), which is based on a ZYNQ processor (Xilinx, 2011). The ZYNQ processor is equipped with a Processing System (PS), a dual-core Cortex-A9 processor, and Programmable Logic (PL) resources equivalent to an Artix-7 FPGA. First, for the ONN on-chip learning architecture, ONN digital design is implemented using PL resources as in Abernot et al. (2021) and is controlled by PS to allow the integration of learning algorithms in PS (see Figure 4).

Communication between PS and PL uses the AXI4-Light parallel communication protocol. We use PS as master and PL as slave such that when PS receives external pattern and command, it controls the digital ONN in PL. If PS receives an external learning command, the Master updates weights following the learning rule and sends weights to the digital ONN in PL. If PS receives an external inference command, PS sends the pattern to the ONN and receives the ONN output after inference. AXI4-Light communication accesses four 32-bit AXI4 registers to send and receive information. The latency of weights transmission, for a given ONN size, depends on the weight precision and the number of weights to fit in a 32-bit register.

The learning process starts when PS receives an external learning command in parallel with an input pattern. It engages the update of the weights on PS following the implemented learning rule before sending the updated weights to the digital ONN in PL through the AXI4-light bus. Note, during weight update, ONN is in reset mode. Once the weight update is over, ONN comes back in inference mode and informs PS that the weight update is done. The inference process starts when PS receives an input pattern with an inference command, such that PS transmits the input pattern through AXI4-Light to the digital ONN in PL, the digital ONN infers, and it sends back its output pattern to PS through the AXI4-Light. Note, an additional command performs a reset of the weights to zeros if necessary.

#### 2.3.3. Evaluation

Here, we study the compatibility of HNN learning rules to ONN on-chip learning for pattern recognition and implement the compatible learning rules in our digital ONN on-chip learning architecture. We evaluate the performances of our architecture with the implemented learning rules through three metrics, resource utilization, capacity, and latency.

We analyze the resource utilization of our ONN on-chip learning architecture as it determines the cost of implementation of our solution in hardware. In Abernot et al. (2022b), authors showed that resource utilization increases drastically from off-chip to on-chip learning for a 15-neuron ONN. In this work, we go beyond and study the scalability of the on-chip learning architecture for larger ONN sizes.

Memory capacity is defined by the number of patterns a network (HNN or ONN) can correctly learn and retrieve. It can be evaluated by learning patterns in the network and verifying if the network retrieves the correct training pattern when one of the training patterns is presented. However, we believe it is also necessary to verify if the network can retrieve the correct training pattern from corrupted input information, corresponding to none of the training patterns, to evaluate the robustness to noise. In this work, we evaluate the capacity of N-neuron HNN and ONN networks trained with up to *N* random training patterns, by testing with corrupted input patterns generated from training patterns with up to *N*/2 flipped pixels, represented by the hamming distance. Note, an inference cycle is performed for each input pattern. Also note, the size of the network, as well as the correlation between the training patterns, impact the capacity of the network, so we perform 100 trials for each configuration. We first evaluate HNN capacity on Matlab to validate Hebbian and Storkey learning rules for three HNN sizes (25, 50, and 100 neurons), then we implement Storkey and Hebbian in the on-chip learning architecture to extract the real capacity metric for a 25-neuron ONN because the resource utilization limits the ONN size. A test flow is set up and automatized for testing the digital ONN on-chip learning architecture using Matlab to send commands and patterns to the system through a UART communication protocol (see Figure 4).

We measure the latency of the 25-neuron ONN for on-chip learning. The latency is divided into three parts, the ONN computation latency, the weight computation latency, and the transmission latency. The ONN computation latency is by default stable no matter the weights and size of the network, so we expect it to stay stable. The weight computation latency mainly depends on the learning rule and computation complexity of the learning rule. And the transmission latency depends on the weight precision and the network size.

## 3. Results

This section presents results obtained with both HNN on Matlab and ONN on FPGA. First, we explain the choice of the most suitable learning rules to implement for ONN on-chip learning. Then, we test the learning rules with ONN on-chip learning constraints in Matlab to study the impact of the weight precision on the HNN capacity and decide which weight precision to apply to the digital ONN design. After, we implement the learning rules in our digital ONN on-chip learning architecture and report on resource utilization, capacity, and latency of our solution for various weight precision.

### 3.1. Learning rules for ONN on-chip learning

In this work, we focus on local and incremental unsupervised learning algorithms introduced for HNNs to be compatible with other ONN implementations. In particular, Tolmachev and Manton (2020) recently surveyed HNN unsupervised learning rules for pattern recognition and studied the impact of weight symmetry, 0-diagonal, and incrementality on HNN pattern recognition capacity. In this work, we consider the various learning rules from Tolmachev and Manton (2020) as potential candidates for ONN on-chip learning and investigate which ones are best suited for ONN on-chip learning (see Table 1). In Tolmachev and Manton (2020), authors show that iterative rules, requiring learning each pattern for more than one iteration (Diederich and Opper, 1987; Krauth and Mezard, 1987; Gardner, 1988) have better precision than other non-iterative learning rules, however, they are often not incremental, making them not suitable for on-chip learning implementation, as shown in Table 1. Table 1 highlights that, based on the learning rules from Tolmachev and Manton (2020), there are only two unsupervised learning rules which satisfy the ONN on-chip learning constraints, Hebbian and Storkey. Storkey learning rule is known to have better capacity than Hebbian, while requiring more computation. The weights update computation *W*_*ij*_ between neuron *i* and neuron *j*, in a network of *N* neurons to learn a novel pattern *ϕ* with Hebbian learning rule is


(2) $$\begin{array}{l} W_{ij}=W_{ij}+\frac{1}{N}\phi_{i}\phi_{j} \end{array}$$

And with Storkey learning rule is


(3) $$\begin{array}{l} W_{ij}=W_{ij}+\frac{1}{N}\left(\phi_{i}\phi_{j}-\phi_{i}h_{ji}-h_{ij}\phi_{j}\right) \end{array}$$

with *h*_*ij*_ a local field computed with


(4) $$\begin{array}{l} h_{ij}=\overset{N}{\underset{k=1}{\sum }}W_{ik}\phi_{k} \end{array}$$

For the rest of the paper, we implement both Hebbian and Storkey learning rules in our digital ONN on-chip learning architecture.

**Table 1:** HNN learning rules features.

**Learning rules**	**Weight symmetry**	**Zero- diagonal**	**Local**	**Incremental**
Hebbian	x	x	x	x
Storkey	x	x	x	x
Diederich Opper I		x	x	
Diederich Opper II			x	
Gardner		x	x	
Krauth Mezard			x	
Pseudo-Inverse	x	x		

### 3.2. Incremental learning with HNN on Matlab

We study the impact of weight precision on HNN accuracy for various HNN sizes. In particular, we analyze the capacity of HNN trained with Hebbian and Storkey for three HNN sizes, 25, 50, and 100 neurons, as well as for five weight precision, 2, 3, 4, 5 bits, and full precision.

**Figure 5:**
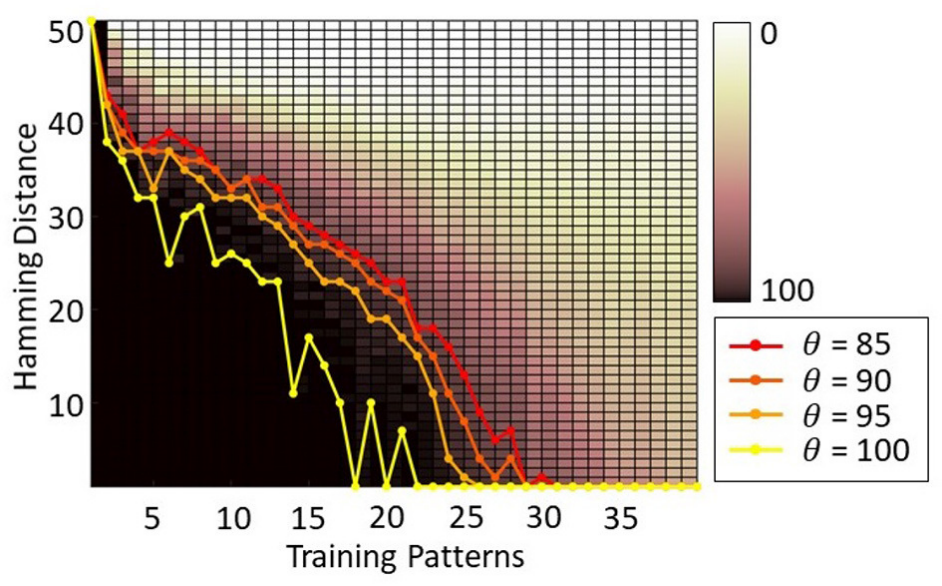
Capacity of a 100-neuron HNN trained with Storkey with 100 training patterns tested with corrupted input patterns with different hamming distances (1 up to 50 flipped pixels) with the training patterns. The capacity lines represent for each number of training patterns the maximum hamming distance of corrupted input patterns supported by the network, such that the network successfully associates the corrupted input pattern with a training pattern for at least *θ* trials over 100.

Figure 5 shows the HNN capacity for a 100-neuron HNN trained with Storkey with 1 up to 100 training patterns and tested for 100 trials with corrupted input patterns with 1 up to 50 hamming distance. A black pixel represents that over the 100 trials, for a given configuration, all tests were successful, while a white pixel points out that none of the tests were successful. The capacity lines highlight, for each number of training patterns, the maximum hamming distance of corrupted input patterns supported by the network, such that the network successfully associates the corrupted input pattern with a training pattern for at least θ trials over 100, with θ = {85;90;85;100}. Then, to simplify the readability of our results, we choose to represent only the capacity lines for one value of θ. We choose θ = 90 to have results representative of a majority of cases and to allow some error tolerance.

**Figure 6:**
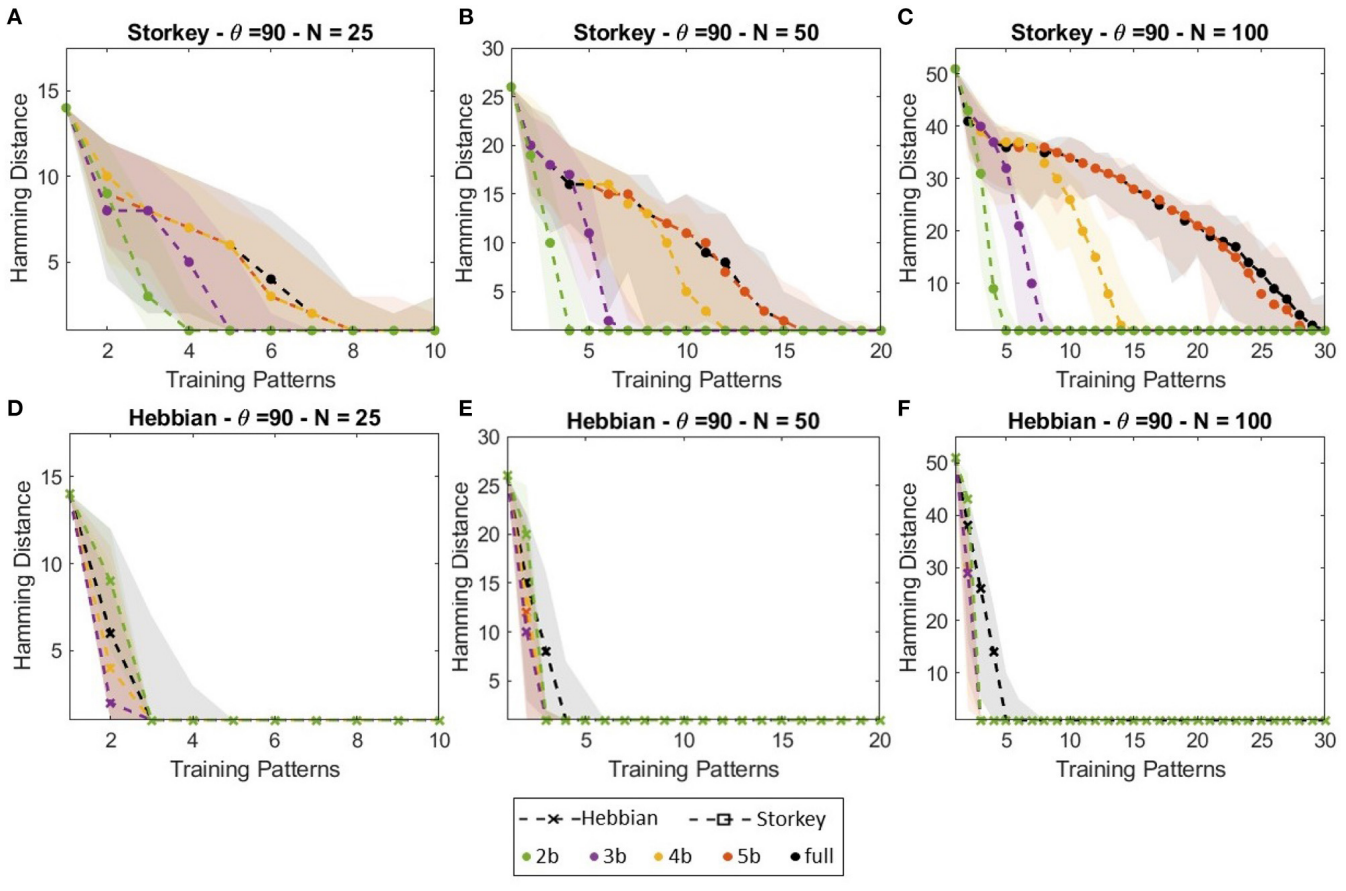
Capacity of HNN networks of **(A, D)** 25 neurons, **(B, E)** 50 neurons, and **(C, F)** 100 neurons trained with **(A–C)** Storkey, or **(D–F)** Hebbian with various weight precision. The capacity is represented, for each network size, for each learning rule, and each number of training patterns, by the maximum hamming distance of corrupted input patterns supported by the network, such that the network successfully associates the corrupted input pattern with a training pattern for at least *θ* = 90 trials over 100 (90%).

Figure 6 shows the HNN capacity lines for θ = 90 for the Hebbian and Storkey learning rules for the different weight precision and network size. Figure 6 also plots the error bounds for each weight precision configuration. Figure 6 first highlights the difference in precision and capacity between Storkey and Hebbian learning rules. HNN trained with Storkey can retrieve a larger number of training patterns when initialized with more corrupted input patterns (patterns with larger hamming distances), thus HNN trained with Storkey shows better capacity than HNN trained with Hebbian for all weight precision configurations. Then, Figure 6 displays that for Storkey learning, using 5-bit weight precision, HNN obtains a similar capacity than considering full weights precision. Note, the impact of reducing weight precision to 4-, 3-, or 2-bit precision depends on the network size. The larger the network is, the more impact the reduction of the weight precision has on the network capacity.

### 3.3. On-chip learning with digital ONN on FPGA

After selecting suitable learning rules and studying their efficiency for HNN on Matlab, we implement Hebbian and Storkey learning rules in our digital ONN on-chip learning architecture and consider three weight precision with 3-, 4-, and 5-bit precision to study the impact on the resource utilization, capacity, latency, and power consumption.

#### 3.3.1. Resource utilization

**Figure 7:**
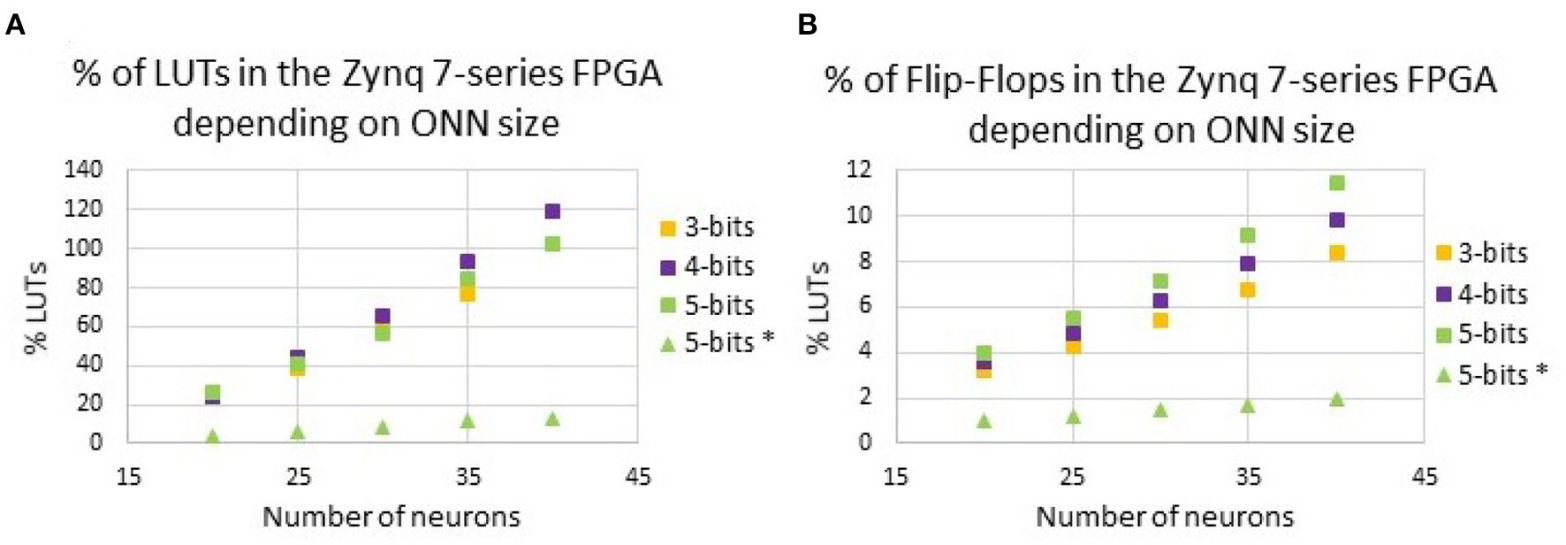
Resource utilization for various ONN sizes for various weight precision with **(A)** LUTs, and **(B)** Flip-Flops. We compare with the previous digital ONN with random hard-coded weights in a 5-bit precision (5 bits*).

First, we report on ONN resource utilization. From Abernot et al. (2022b), we know that for a small 15-neuron scale ONN, re-programmable synapses utilize a large number of resources, in particular Look-Up-Tables (LUTs). In the proposed architecture, a large number of LUTs are used as reconfigurable memory of the weight matrix, so due to the fully-connected ONN architecture, the number of synapses increases following *N*(*N*−1) for *N* neurons, and so the number LUTs also increases. Figure 7 highlights the LUTs and Flip-Flops utilization for ONN with 20 up to 40 neurons with and without on-chip learning.

To limit the impact of re-programmable synapses, we analyze the impact of reducing the weight precision on resource utilization. In Figure 7, we report on the number of Look-Up-Tables (LUTs), as well as the number of Flip-Flops (FFs) necessary for our digital ONN implementation, for 3-, 4-, and 5-bit precision. As mentioned previously, in the proposed architecture, a large number of LUTs are used as reconfigurable memory of the weight matrix. Thus, we expect the reduction of the weight precision to also reduce LUTs utilization. However, Figure 7 indicates that for some ONN sizes, reducing the weight precision does not reduce the number of LUTs. For example, for the 35-neuron ONN, the number of LUTs is larger for the 4-bit precision than for the 5-bit precision. We believe it depends on the configuration of the FPGA, which provides fixed-size LUTs. Additionally, the reduction of the weight precision from 5 to 3 bits does not significantly reduce the resource utilization as expected, limiting the ONN size for on-chip learning implementation. With our solution, we can implement an ONN with up to 35 fully-connected neurons with re-programmable synapses. Next, we consider a 25-neuron ONN to report on its capacity and latency.

#### 3.3.2. Capacity

**Figure 8:**
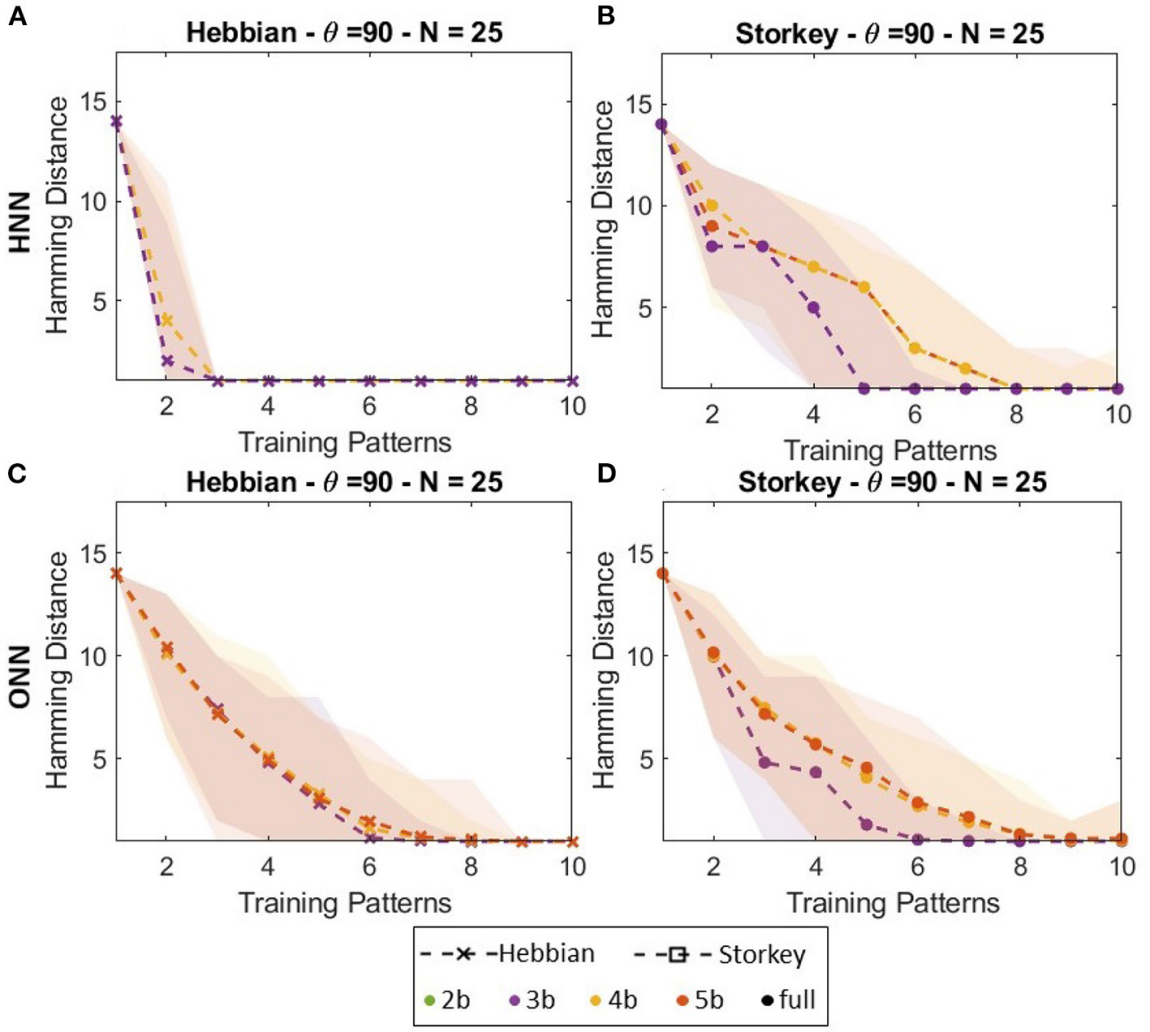
Capacity of **(A, B)** HNN and **(C, D)** ONN networks of 25 neurons trained with **(A, C)** Hebbian or **(B, D)** Storkey.

Figure 8 presents capacity lines obtained for a 25-neuron digital ONN trained on-chip with both Hebbian or Storkey for three different weight precision (3, 4, and 5 bits) compared with HNN trained with the same configuration. Figures 6B, D show that for Storkey on-chip learning, HNN and ONN have similar capacities. However, considering Hebbian learning, Figures 8A, C demonstrate ONN has a better capacity than HNN. Figure 8 also shows less ONN capacity variations depending on the weight precision than HNN capacity. These are unexpected as were not observed in previous configurations, but this is, to the best of our knowledge, the first large-scale capacity tests performed with the digital ONN. We believe the difference between HNN and ONN trained with the Hebbian learning rule might come from the difference in the system dynamics between HNN and ONN. Classical HNN can only take two state values, −1;1, because of the sign activation function. However, the ONN activation function allows it to take multi-state or continuous values during dynamical evolution. Thus, even if an ONN trained with binary patterns will stabilize to binary phase states 0°;180°, the activation function, which is difficult to derive, allows non-binary phase states during phase dynamics. We believe that the phase dynamics of the ONN evolve slowly from a corrupted input pattern to the correct training pattern, while the sharp HNN activation function may evolve too fast, reaching a wrong training pattern. HNN may require more precise weights, as with Storkey, to take the correct decision, while the ONN can still evolve to a correct training pattern even with less precise weights. However, we believe it requires additional investigation to draw conclusions. It is important to note that our architecture enables incremental on-chip learning of a digital ONN design with two different learning rules, Hebbian and Storkey, for pattern recognition tasks.

#### 3.3.3. Latency

Finally, we report on training and inference latency for a 25-neuron ONN working at *F*_*osc*_ = 187.5 KHz. Concerning inference, we measure input pattern transmission latency from PS to PL, ONN computation latency in PL, and ONN output transmission latency from PL to PS. Table 2 shows that ONN inference takes around two to three oscillation cycles to compute, similar to the solution with off-chip learning (Abernot et al., 2021). Then, the transmission of ONN input and output takes 27μs which is 1.5 times higher than the ONN computation. Note, increasing the ONN size will also increase the transmission latency as the information to transmit will be larger, while the ONN computation should stay stable. Thus, the architecture increases the inference latency compared to off-chip learning solutions because of information transmission from PS to PL, and reversely.

**Table 2:** Measurements of latency for ONN training and inference with ONN oscillation frequency *F*_*onn*_ = 97.7*KHz* and PS clock frequency *F*_*PS*_ = 667*MHz*.

**This work**				**Abernot et al. (2022b)**
**Weights**	**3 bits (**μ**s)**	**4 bits (**μ**s)**	**5 bits (**μ**s)**	**5 bits**
**Training**				
Hebbian learning	55			33 μs
Storkey learning	210			77 μs
Weight precision	140			NA
Weight transmission	18	71	175	86 μs
Total Hebbian	213	266	370	119 μs
Total Storkey	368	421	525	163 μs
**Inference**				
Input transmission	9			NA
ONN computation	17			NA
Output transmission	18			NA
Total	44			NA

Concerning training, we differentiate the latency into three steps, one to perform the training algorithm in PS, another to rescale weights to the corresponding weight precision, and finally to transfer weights from PS to the ONN in PL. Table 2 highlights that Storkey requires more computation time than Hebbian. This is because Storkey requires more computation than Hebbian, see Equations (2) and (3), and PS performs sequential processing. Then, weight transmission increases drastically with the increase of the weight precision and the number of neurons. Reducing the weight precision has an important impact to reduce transmission latency because we use AXI4-Lite with 32-bit parallel transmission.

Our solution, for a network of 25 neurons, allows computing Hebbian in 55 *μ*s, and Storkey in 210 *μ*s. Additionally, to allow reducing weight precision to 3, 4, or 5 bits, additional treatment is necessary, taking 140 *μ*s. Then, transmission time depends on the weight precision taking between 18 and 175 *μ*s. In total, training a fully-connected ONN, configured for 5-bits signed synapses, with a novel training pattern takes 370 *μ*s with the Hebbian learning algorithm and 525 *μ*s with the Storkey learning algorithm. Thus, because Hebbian and Storkey have similar precision in the digital ONN design, it can be more of interest for a system with high time constraints to implement Hebbian rather than Storkey on-chip learning.

#### 3.3.4. Power consumption

We extract the estimated post-place and route power consumption of our digital ONN with re-programmable synapses on Vivado considering the xc7z020-1clg400c target, and we compare it with the digital ONN implementation without the re-programmable synapses (Abernot et al., 2021) and with other fully-connected ONN implementations (Jackson et al., 2018; Bashar et al., 2021; Delacour et al., 2023b). We compute the energy per neuron per oscillation by considering an ONN computation time of three oscillation cycles. Table 3 highlights that the digital ONN with re-programmable synapses requires slightly more energy per oscillation than the digital ONN without re-programmable synapses (Abernot et al., 2021), certainly because of the additional LUTs resources necessary for the on-chip learning. Also, both digital ONNs are in the same energy per oscillation range as the analog ONN implementation in Bashar et al. (2021) as they operate at a lower frequency than the other implementations (Jackson et al., 2018; Delacour et al., 2023b). Using a higher ONN frequency could reduce the computation time, ultimately reducing the energy per computation and oscillation, however, the digital ONN frequency is currently limited by the FPGA.

**Table 3:** Comparison of the digital ONN with re-programmable synapses with other fully-connected ONN implementations.

	**Jackson et al. (2018)**	**Bashar et al. (2021)**	**Delacour et al. (2023b)**	**Abernot et al. (2021)**	**This work**
Neurons	100	30	16	60	25
Power	303 mW	1.76 mW	160 *μ*W	20 mW	10 mW
Frequency	1 GHz	45 kHz	1 MHz	187.5 kHz	187.5 kHz
Energy/osc	0.3 pJ	1.3 nJ	10 pJ	1.78 nJ	2.13 nJ

## 4. Discussion

This paper studies possible algorithms and provides an implementation to perform continual on-chip learning with a digital ONN design for pattern recognition. It highlights that HNN unsupervised learning algorithms are compatible with ONN on-chip learning only if they satisfy two constraints on the weight matrix, the symmetry and the 0-diagonal, and two additional constraints on the learning algorithm, locality, and incrementality. This work evaluated seven state-of-the-art unsupervised learning rules developed for HNN (Tolmachev and Manton, 2020) and defined two of them to be compatible with ONN on-chip learning, Hebbian and Storkey. Both Hebbian and Storkey learning rules exhibit similar capacity results when implemented in the proposed architecture to perform on-chip learning on a 25-neuron ONN, making them both suitable for continual ONN on-chip learning.

The proposed architecture takes advantage of a Zynq processor (Xilinx, 2011) equipped with both PS and PL resources to implement a fully-connected digital ONN introduced in Abernot et al. (2021) with re-programmable synapses in PL, and execute the unsupervised Hebbian and Storkey learning algorithms in PS. The architecture was first introduced in Abernot et al. (2022b) for a small-size ONN with 15 neurons, while this work evaluates the scalability of the architecture. First, it is important to highlight that the solution does not require many changes from the first digital ONN design, making it easy to adapt and install. The main scalability limitation of the architecture is due to the digital ONN re-programmable synapses which demand a large number of LUTs, even with reduced weight precision, limiting the ONN size up to 35 fully-connected oscillators while the digital ONN without re-programmable synapses could reach hundreds of fully-connected neurons (Abernot et al., 2021). Another limitation of the architecture is the latency induced by the separation between ONN learning and computation in PS and PL. On one side, PS allows to implement and compute a large panel of unsupervised learning algorithms, executing them sequentially with a fast frequency of *F*_*ps*_ = 666 MHz. On the other side, it generates latency to transmit the weights from PS to PL, increasing with the ONN size. An alternative solution is to implement the training algorithms using the parallel properties of PL resources to provide fast training and remove the transmission latency. However, we believe it would utilize additional PL resources, including LUTs, which are already limited. Another solution is to use other communication protocols than AXI-Lite between PS and PL, such as AXI-stream which provides more parallel transmission. Overall, our solution permits to train a 25-neuron ONN in hundreds of microseconds, between 350 and 550 *μ*s which is the first solution to perform ONN on-chip learning.

Future work will first explore alternative solutions to try to overcome the current limitations of the ONN on-chip learning architecture. Furthermore, the next developments will focus on possible applications with the ONN on-chip learning architecture. The digital ONN design has already been used for sensor data treatment in various applications, like interfacing with a camera for image recognition (Abernot et al., 2021) or using proximity sensor data to perform obstacle avoidance (Abernot et al., 2022a), so we are confident on the integration of our architecture with different sensors. Possible applications for the digital ONN on-chip learning architecture could be in the robotics domain where real-time continual learning is often necessary, and where the digital ONN design already showcased good performances (Abernot et al., 2022a). For example, navigation, in the context of mobile robots, is a complex task depending on the environment, where continuous learning is necessary to adapt to evolving situations. A first proof of concept of two pre-trained cascaded ONNs performing obstacle avoidance from proximity sensors was shown in Abernot et al. (2022a). Though in Abernot et al. (2022a), the pre-trained ONNs are capable of finding a novel direction using information from 15 proximity sensors whose configurations are used to define the training patterns. However, if we consider an obstacle avoidance application using more sensor information than 15 proximity sensors, it becomes impossible to define all possible training patterns before inference. Using ONN on-chip learning allows training the ONN continuously through time depending on the environmental configuration given by the sensory information. Thus, we believe that using the ONN on-chip learning architecture can be beneficial in the case of applications with large-scale inputs where all possible configurations can not be anticipated. A first idea was proposed recently to perform real-time ONN on-chip learning for an obstacle avoidance application using the proposed architecture (Abernot et al., 2023b), however, a demonstrator is yet to be developed.

## 5. Conclusion

This work analyses unsupervised learning rules for Oscillatory Neural Network (ONN) learning for pattern recognition tasks, and in particular for continual ONN on-chip learning. We evaluate the adaption of unsupervised learning rules developed for Hopfield Neural Networks (HNNs) for ONN on-chip learning and show that Hebbian and Storkey learning rules are both suitable for ONN on-chip learning. Additionally, we propose an architecture capable of performing ONN on-chip learning using a digital ONN implementation with various unsupervised learning algorithms. It uses a Processing System (PS) of a Zynq processor to implement the learning algorithms and Programmable Logic (PL) resources to implement the digital ONN. We point out that the architecture limits the network in size, with up to 35 neurons, due to the large resource utilization. Also, with the proposed architecture, learning and inference latency increase with the network size, which can become a limitation for time-constrained systems. Our current solution can train a 25-neuron ONN on-chip in hundreds of micro-seconds, between 350 and 550 *μ*s. This is, to the best of our knowledge, the first solution to perform ONN on-chip learning with unsupervised learning algorithms for pattern recognition. We believe it can be useful for investigating novel ONN learning algorithms and applications such as reinforcement learning for robotic applications.

## Data availability statement

The raw data supporting the conclusions of this article will be made available by the authors, without undue reservation.

## Author contributions

AT-S motivated the project and experiments. MA performed the survey on the learning rules, performed the HNN tests, developed the on-chip learning architecture, and performed the measurements. AT-S and NA were involved in the discussion and editing of the manuscript and provided valuable inputs at multiple stages of this work. All authors contributed to the article and approved the submitted version.
